# Chloroform Its Effects, &c.

**Published:** 1855-06

**Authors:** P. A. Allaire

**Affiliations:** Aurora, Ill.


THE NORTH-WESTERN

### MEDICAL AND SURGICAL JOURNAL.

NEW SERIES.

VOL. IV. JUNE, 1855. NO. 6.

### ORIGINAL COMMUNICATIONS.

ART. I.—*Chloroform. Its Effects, &c.* 2nd Article. By P. A. Allaire, M. D.

The use of Chloroform by inhalation was noticed at some length in a former article in the last number of this Journal, to finish the subject it remains to speak of its use by the stomach and locally.

By the Stomach, Chloroform is used chiefly as an Anodyne and anti-Spasmodic. It possesses, however, when thus given, a slightly stimulant effect, it also acts on a soporific, but this seems to be a result of its anodyne quality. Some writers have described it as producing decided sedative effects when given in this way; this in fact, seems to be the common opinion, but it is not in accordance with my experience of its influence. I believe this idea to have arisen from the fact of its being so decided a sedative when used by inhalation.

That it does not, when given by the Stomach act as a sedative, does not depress the nervous system, is well proven by the following extract from a case of Delirium Tremens described in Braithwaite's Retrospect, No. 30, page 42: "It was now four days since he had had any sleep He was so hoarse as to be unintelligible from incessant talking and vociferating; eyes congested; pupils much contracted; tongue dry, and protruded with difficulty; urine scanty; tremors of the hands and arms with subsultus tendinum. His strength was evidently failing, and he no longer struggled to leave the bed; the pulse had become smal er and more rapid He was not sinking from want of stimulus or of nourishment, for both had been given in sufficient quantity, but death was impending for want of sleep, and opium had been pushed to the uttermost. Under these most unpromising circumstances I recommended Chloroform to be given internally; a drachm was immediately given, and repeated in an hour; soon afterwards he slept for a few minutes; a third dose also containing ʒi was now given and soon he slept soundly. The next day he was free from every symptom of the disease." Here was a well marked case, where the patient was sinking from pure nervous exhaustion, and yet the medicine was given in the largest doses and at the shortest intervals, yet he did not continue to sink; on the contrary he rallied and slept. This shows an active anodyne, and mild stimulant as effects of the remedy.

The dose for an adult is from 10 drops to ʒi given every 2, 3 or 6 hours. What the effect might be on the Stomach or nervous system, if these doses were much increased has not yet been ascertained. Patients that I have given forty and fifty drop doses to, for pain of a neuralgic character, and who were free from any other nervous symptoms, have been unwilling to increase the amount in consequence of a peculiar feeling of lightness which it caused; no sleepiness was produced, but with the sensation of lightness was a calm comfortable ease. In the Stomach was a slight warmth for a short time after each dose The lesser dose does not increase the heart's action or add to the natural warmth of the skin; the larger doses however, do, but very slightly. None of the secretions are increased or diminished during its use, nor does it produce any constipating effect. It seems to be soon removed from the system by the lungs, yet the soothing influence produced by it frequently continues for some time, sleep having lasted eight or ten hours after its exhibition by the stomach in cases of Delirium Tremens. Chloroform has not been used by the stomach in the treatment of a large number of diseases; Delirium Tremens, Tetanus, Hypochondriasis, Neuralgia, some forms of Hysteria, and the Hyperaesthesia Cerebri present in some forms of fever are the chief, and in these it has been had resourt to rather as a last resort, and generally when all the other usual remedies had failed; yet under these most unfavorable circumstances. it has not disappointed the hopes of the practitioner, as many cases now published will testify. There would seem to me to be a larger field for the powers of this remedy, and that it ought to be placed with other anodynes and anti-spasmodics as suitable in many other forms of disease. Every practitioner knows how often he finds it necessary to exhaust the list without finding that effect from any one which is precisely suited to the condition of the case in hand. Might not Chloroform, then, in these circumstances be relied on, either alone or in combination with some other article of known powers, with Morphine, Camphor, Valerian, Asafœtida, Lupulin, &c.

There is a form of nervous exhaustion seen occasionally during the progress of fevers and some other debilitating diseases, which is very distressing to the patient and alarming to his friends. The prominent symptoms are a hurried respiration, enfeebled and quickened pulse, and generally a cool and moist skin, though. this may be hot and dry in Malarious fevers. The patients are generally females and their great suffering seems to be caused by a deficient supply of oxygen to the blood, as they constantly demand fresh air and the use of the fan. The air in those cases seems to hurry in and out of the bronchi without entering and distending the air cells, in fact auscultation gives a very imperfect respiratory murmur. If this condition is not soon relieved, more or less congestion of the lungs results. I have used the Chloroform in doses varying from ten to thirty drops here, with great apparent advantage, while the ordinary stimuli and anti-spasmodics seemed to have little effect, but as it was not trusted to alone its real utility cannot be settled; the patients, however, always expressed themselves as greatly relieved. Some cases of Neuralgia treated with Chloroform given inwardly may be found reported by me in this Journal about three years since, many other cases of like character have been since published. The judicious physician will not be likely to make errors in giving this medicine internally if he remembers that its effects are mainly anodyne, and that its effects are most marked where the exaltation of sensibility is the greatest. The following case illustrates its benefits in that singular form of disease known as Hypochondriasis. It is extracted from a paper published by Prof. OSBORNE of Dublin, on the internal use of this agent in this class of diseases: "The third case was that of a farmer, twenty eight years of age. The angles of his mouth were drawn down, his brow generally contracted, and he appeared sometimes as if contemplating suicide, yet he stated that he was in comfortable circumstances, married and with a family. He complained of an inward sinking and sense of depression, so constant and overpowering, that for some months he could not command himself to make any exertion, and had become unable any longer to attend to his business. His bowels were usually torpid, but although he had repeatedly taken purgative medicines with effect, yet he had obtained no further benefit. A careful examination having been made without detecting any disease, he was ordered to take ten drops of chloroform thrice daily, and two asafœta pills every alternate night. At the end of about four days of gradual improvement his countenance had become placid *He confessed that he felt much better;* and in a few days afterwards, feeling a strong desire, and also a capability of resuming his ordinary vocations, he went home." To hear a hypochondriac say he is better, is good evidence that he is improving. These cases are well known as among the most troublesome that come under the care of the physician. I have lately treated a case of nervous irritation with chloroform which illustrated its anodyne effects finely. The patient, a man about fifty-five years of age, has had for the last fifty years a splinter of wood in his right hand; about a year since a piece of the splinter was removed—a small piece still remains. When the spot where this is located becomes irritated from any cause, the whole nervous system becomes irritable, and the nervous cords of the limbs painful; are tender on pressure, especially those in the arm leading to the effected part. The nerves on the limbs on the opposite side are less tender and painful, but the point in the opposite hand which corresponds to the situation of the foreign body is quite tender. This attack had lasted several weeks when first seen by me, all the symptoms gradually increasing in severity; he had also lately had another attempt made at extraction of the foreign body, which was unsuccessful. For the last two or three days he has not slept over an hour or two—generally a short sleep of about half an hour in the night. He is much worn. Appetite fair. After giving morphine, valerain, asafœta, &c., a fair trial, I directed him to take ten drops of chloroform every three hours, and increase the dose gradually. He commenced in the morning after a sleepless night, or if there was a momentary sleep, he awoke frightened and anxious. He declared the nervous uneasiness to be indescribable. The first night after using the medicine there was not more than half an hour's good sleep, but he described his feelings as those of ease and comfort. The second day he took doses of twenty drops every three hours; and the second night after commencing the medicine he slept well—declaring that he had never before appreciated the value of sleep. From this time his recovery was steady under the use of chloroform. Sleeplessness is the great source of danger in Delirium Tremens, and in some other diseases where Hyperæsthesia is a permanent symptom; hence the necessity oftentimes of treating this symptom mainly, and the value of any remedy on which we can rely. Chloroform is most valuable in that form of delirium tremens known as sthenic in which opium is not generally admissable in large doses here; where there is active congestion of the brain, and we cannot procure sleep by the careful use of purgatives, cold to the head, and perhaps the loss of a little blood from the part we may use the chloroform with hope of a good result, as also in the asthenic form where opium has been pushed to its utmost limits as shown by a dry tongue and contracted pupils.

Chloroform may be exhited in any mucilage and forms a permanent mixture when the proportion of the medicine is not too large. The sweet taste which it gives may be covered if desired by a bitter. "Another mode of avoiding the pungency of chloroform is by giving it in combination with tinctures, as it is soluble in Alcohol and remains dissolved in proof spirit. The following is a specimen of this kind of formula, and is peculiarly grateful to the taste and susceptible of various additions and alterations according to the nature of the cases:

℞ Chloroform.
Tinct. Zinziber ace ℥.
Spt. Ammon. Aromatic ʒ.

The following I have found a good formula where a mild diffusable stimulant and anodyne effect was needed. It was to be shaken before use, as it partially separates on standing.

℞ Tinct. Opii
Tinct. Camph. and Chloroform equal parts, mix.

These proportions may be varied to suit the case. Locally, Chloroform has been much used, either alone or as an ingredient in anodyne and stimulant embrocations. When thus applied it produces no perceptible effect on the circulation or secretions but to the skin it acts as a stimulant and rubefacient, the local anodyne effect with the stimulation of the surface is often sufficient to relieve and even entirely remove many painful local affections. The cases in which such a remedy would be likely to prove useful will suggest themselves to the practitioner.

If the vapour of Chloroform is kept in contact with any part for a certain time, it produces a decided anodyne or even local anæsthetic effect, and when this effect has subsided, the part retains all its natural powers and healthful functions. Some very interesting cases illustrating this mode of using Chloroform and describing an ingenious instrument for effecting this purpose, will be found in a paper by Dr. Hardy, and republished in Braithwaite, part 27, page 278. The cases are Cancer of Uterus, Dysmenorrhœa, Ulcerated and irritated Uterus and irritable breast.—The following case and description of the instrument are from Dr. H.'s paper:

Case 3. This woman has long suffered from Dysmenorrhœa, Leucorrhœa and excoriation of os and cervix uteri. "On the 15th September she again consulted me on account of severe lumbar and uterine pain, which on the day previous was so excruciating and accompanied with so much pain in her breast, that she thought it impossible she could have borne it. This day the pain in the breast was better, but the pain in the back and in the pubic and uterine regions is as great as yesterday. The uteras was tender to the touch, but no abrasion was now discovered with the speculum. The vapour of chloroform locally applied by the anaesthetic douche had the most immediate and happy effects. In no case that I have met with did relief so soon succeed to its use.—Not more than a minute could have passed from commencing its application, when the patient expressed herself relieved from, first the lumbar pain. and next that in the pubic and uterine regions. After I had withdrawn the instrument, for some time there remained a sensation of warmth, of a very agreeable nature, and a feeling of strength was experienced in the back. Altogether, she said, for months she had not been so free from pain or so comfortable. Judging from the expression of countenance, the change so immediate from suffering to freedom of pain was most remarkable. There was no return of it until about eight o'clock in the evening, the application having been made between twelve and one o'clock; but it was then so trivial that she did not think anything of it. The next day two or three jets of the douche quite removed every trace of uneasiness."

The relief procured from the chloroform used in this way is often as permanent as by the internal use of opiates, the great advantage being in the saving of nervousness, loss of appetite, constipations, &c., which follows their use in many constitutions. The apparatus by which chloroform in vapour is applied is so simple, that with ordinary ingenuity any practitioner may make one for his own use: "It consists in a small metallic box or chamber; this holds the sponge which is to contain the chloroform; this box may be made of tin with a lid to shut tight, for removing the sponge when needed; there must also be in the box two openings opposite each other; to one of which is attached a gum elastic bottle with a valve admitting air, to the other a pipe of such size and length as is necessary to carry the vapour to the part. By pressing the elastic bag, the air is forced through the sponge and out of the pipe charged with vapour." The vapour may be kept in contact with flat surfaces by confining it under a piece of oiled silk or paper; and in cavities by closing the orifice around the pipe.

The minor operations may many of them be rendered painless by the local application of chloroform vapour; a common method of using it for this purpose is to cover the part with lint or cotton saturated with chloroform, and then force a current of air through the lint on the part to be rendered insensible; this means is imperfect, requiring often much time and frequent renewing of the chloroform. In using chloroform topically we should always endeavor to make the application to the seat of disease, though frequently embrocations or liniments will afford much relief to parts which are suffering from sympathy. In neuralgic pain of small extent pure chloroform applied with a cloth and pressed on the part, is very efficacious. The numerous painful afflictions in which it may be applied with benefit I need not here enumerate. It may, however, be useful to append a few formula for embrocations, liniments, &c.

℞ Chloroform.
Gum Camph., equal parts, make a solution.

This is one of the best of "toothache drops," and is often used without the camphor.

℞ Chloroform.

Ol. Olive.

Ol. Terebinth., equal parts, mix.

Used as a stimulating liniment. If applied with heat it is very active; a hot cloth saturated with it should be laid on the part when a visicatory effect is desired. All liniments made with chloroform should contain a fixed oil to prevent its too speedy evaporation.

℞ Chloroform ʒij

Pulv. Gum Camph. ʒj.

Extr. Belladonnæ ʒj.

Ungt. Simp. ℥j.

Rub the ointment and extract together, then add the camphor and lastly the chloroform. This preparation makes a very useful anodyne embrocation.

℞ Chloroform.
Olive Oil.
Lime Water, mix equal parts.

Much superior to the common preparation of lime water and sweet oil as an application to superficial burns.

AURORA, Ill., April, 1855.

